# Supplementary material for: Beryllium Dimer Reactions with Acetonitrile: Formation of Strong Be−Be Bonds
Source: Molecules. 2023 Dec 28;29(1):177. doi: 10.3390/molecules29010177 (PMC10779904; doi:10.3390/molecules29010177)
Supplement: Supplementary file 1 [file molecules-29-00177-s001.zip › molecules-2740914-supplementary.pdf]

Electronic Supplementary Information

Beryllium Dimer Reactions with Acetonitrile: Formation of Strong Be–Be Bonds

Table of contents

Figure S1 The Spin Density Plots of $BeNCCH_3$ at the B3LYP–D3/aug–cc–pVTZ Level of Theory. **S3**

Figure S2 Selected frontier molecular orbitals of $CNBeBeCH_3$ calculated at B3LYP–D3/aug–cc–pVTZ level of theory. **S3**

Figure S3 Contour line diagrams of the Laplacian of the electronic density of the mono beryllium products. **S4**

Figure S4 2D Localized Orbital Locator (LOL) map of the mono beryllium products. **S5**

Table S1 Observed and Calculated Fundamental Frequencies of $BeBeNCCH_3$ Isotopomers in the Ground $^3A'$ State. **S6**

Table S2 Observed and Calculated Fundamental Frequencies of $BeNCCH_3$ Isotopomers in the Ground $^3A_1$ State. **S7**

Table S3 Observed and Calculated Fundamental Frequencies of $CNBeBeCH_3$ Isotopomers in the Ground $^1A_1$ State. **S8**

Table S4 Observed and Calculated Fundamental Frequencies of $CNBeCH_3$ Isotopomers in the Ground $^1A_1$ State. **S9**

Table S5 Observed and Calculated Fundamental Frequencies of $HBeCH_2CN$ Isotopomers in the Ground $^1A'$ State. **S10**

Table S6 Observed and Calculated Fundamental Frequencies of $HBeNCCH_2$ Isotopomers in the Ground $^1A_1$ State. **S11**

Table S7 Calculated Fundamental Frequencies of the cyclic intermediate in the Ground $^1A_1$ State. **S12**

Table S8 NBO Analysis of Products Using the B3LYP–D3/aug–cc–pVTZ Level of Theory. **S13**

Table S9 EDA-NOCV Results at the B3LYP/TZ2P/ZORA Level of Theory for $CNBeBeCH_3$. **S14**

Table S10 AIM Analysis of BeN, BeC and BeBe bonds in Products. **S14**

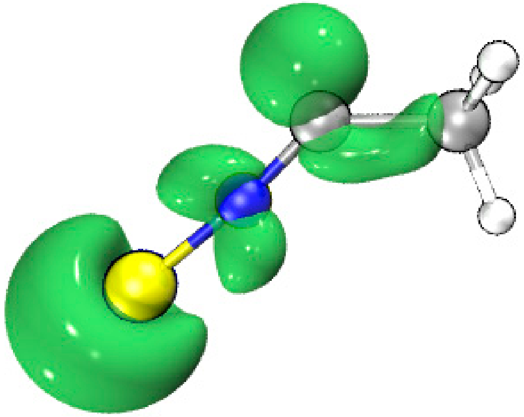

Figure S1 The Spin Density Plot of $BeNCCH_3$ at the B3LYP–D3/aug–cc–pVTZ Level of Theory. The values of Mulliken spins are given in a.u. Isosurface values are 0.01 a.u.

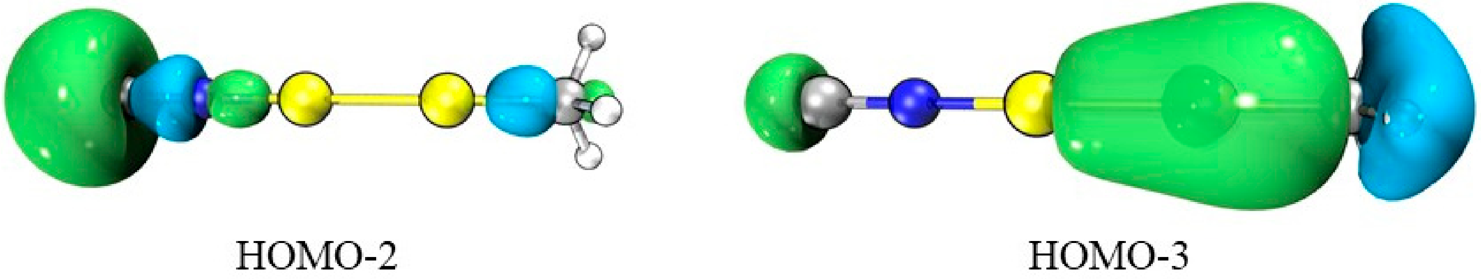

Figure S2 Selected frontier molecular orbitals of $CNBeBeCH_3$ calculated at B3LYP–D3/aug–cc–pVTZ level of theory.

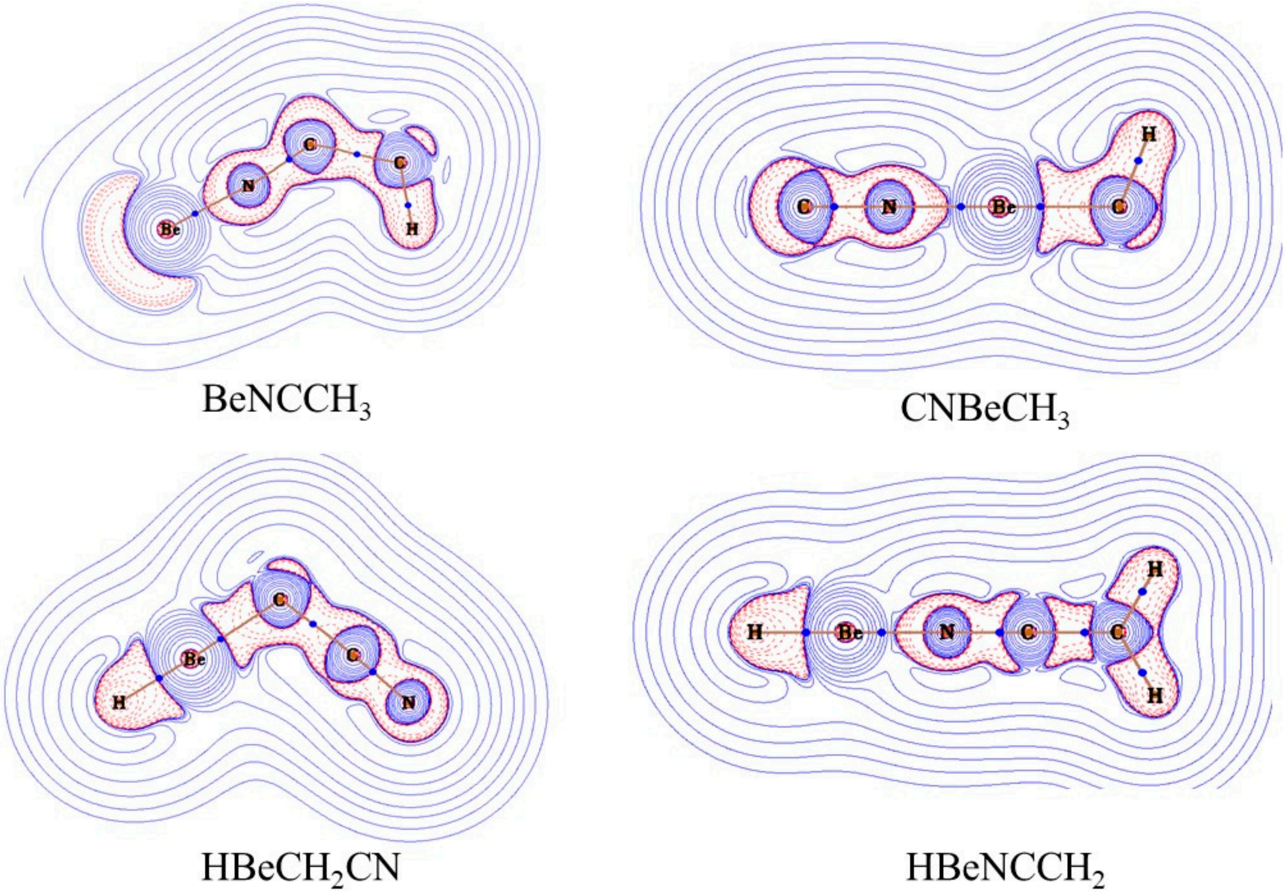

Figure S3 Contour line diagrams of the Laplacian of the electronic density of the mono beryllium products. Blue dots stand for BCPs. The blue solid lines and red dotted lines correspond to values of $\nabla^2\varrho(r) > 0$ and $\nabla^2\varrho(r) < 0$, respectively.

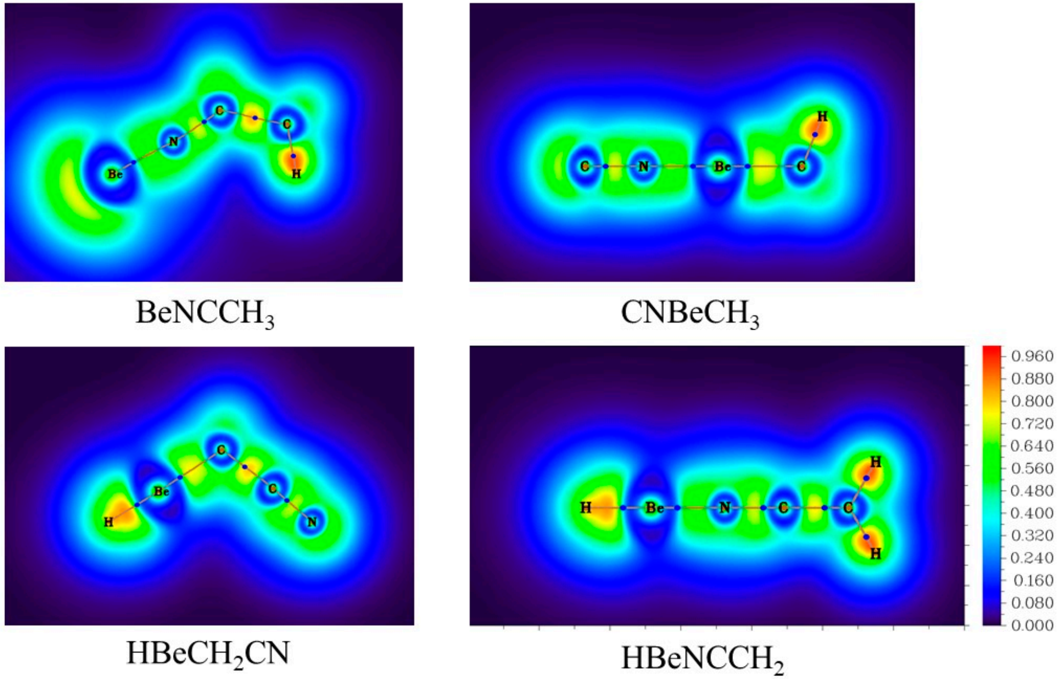

Figure S4 2D Localized Orbital Locator (LOL) map of the mono beryllium products.

Table S1 Observed and Calculated Fundamental Frequencies of $BeBeNCCH_3$ Isotopomers in the Ground $^1A'$ State[a]

Approximate Description	$BeBeNCCH_3$					$BeBeNCCD_3$					$BeBeN^{13}C^{13}CH_3$				
	Obs[b]	Harmonic	Int	Anharmonic	Int	Obs[b]	Harmonic	Int	Anharmonic	Int	Obs[b]	Harmonic	Int	Anharmonic	Int
A′ CH_3 as. str.		3112.3	0	2965.3	0		2303.3	0	2210.1	0		3101.3	0	2941.1	0
A″ CH_3 as. str.		3086.0	20	2954.0	4		2272.6	20	2185.2	10		3077.4	17	2933.5	1
A′ CH_3 s. str.		2953.6	209	2836.4	241		2131.2	57	2070.0	52		2948.2	217	2765.6	241
A′ C–N str.	1927.8	1986.0	1132	1944.9	919	Covered[c]	1984.3	1151	1927.6	873	1889.4	1942.7	1098	1895.0	930
A″ CH_3 twist		1457.7	56	1424.1	44		1108.3	17	1079.1	10		1455.9	59	1422.0	54
A′ CH_3 twist		1449.5	7	1398.3	4		1046.6	20	1027.9	20		1447.5	7	1397.8	6
A′ CH_3 wag		1377.0	8	1342.5	13		1044.1	3	1020.3	3		1367.5	12	1322.4	17
A′ N–Be str.		1081.2	32	1032.4	25		1023.8	5	981.2	5		1069.4	35	1018.2	35
A′ CH_3 bend		1017.1	0	980.9	1		812.1	0	783.7	0		1005.5	1	972.2	1
A′ CH_3 twist	994.1	986.0	581	954.1	501	827.0	812.3	754	790.1	638	981.2	975.1	504	943.1	446
A′ C–C str.		832.1	133	812.0	113		765.1	23	751.4	33		815.7	140	798.4	111
A′ Be–Be str.		458.0	129	449.6	123		444.8	63	432.4	60		452.8	113	438.7	97
A′ CNBe oop bend		334.8	4	333.0	4		325.7	5	312.8	5		331.7	4	320.3	5
A′ CNBe ip bend		326.5	549	319.6	514		320.4	499	301.5	511		323.2	539	304.3	553
A′ NBeBe ip bend		204.2	24	200.8	26		198.6	25	181.7	23		202.9	26	186.0	23
A′ CCN bend		102.9	4	125.5	4		92.1	3	69.8	4		102.4	4	110.2	5
A′ Be–Be oop bend		77.0	19	95.7	2		73.4	18	49.0	0		76.3	19	65.0	3
A′ Be–Be ip bend		75.7	0	75.9	19		65.5	1	40.5	18		75.4	0	42.1	21

[a] Frequencies and intensities are in cm^{-1} and km/mol. The computed frequencies are given at the B3LYP–D3/aug–cc–pVTZ level of theory. [b] Observed in a Ne matrix. [c] Absorptions covered by precursor bands. $BeBeNCCH_3$ has a C_s structure.

Table S2 Observed and Calculated Fundamental Frequencies of $BeNCCH_3$ Isotopomers in the Ground $^3A_1$ State[a]

Approximate Description	$BeNCCH_3$					$BeNCCD_3$					$BeN^{13}C^{13}CH_3$				
	Obs[b]	Harmonic	Int	Anharmonic	Int	Obs[b]	Harmonic	Int	Anharmonic	Int	Obs[b]	Harmonic	Int	Anharmonic	Int
A″ CH_3 as. str.		3097.5	1	2940.7	3		2291.1	1	2209.0	1		3086.6	1	2936.9	2
A′ CH_3 as. str.		3082.4	9	2933.3	5		2279.0	9	2195.8	7		3072.7	8	2929.3	4
A′ CH_3 s. str.		2990.5	37	2868.6	55		2150.4	12	2078.2	14		2986.1	38	2865.2	52
A′ C–N str.	1915.4	1953.5	119	1918.0	107	1911.2	1952.1	124	1909.7	110	1875.4	1915.6	121	1885.8	103
A′ CH_3 bend		1461.1	36	1419.7	32		1129.5	23	1029.2	3492		1459.1	37	1416.3	29
A″ CH_3 bend		1457.0	9	1413.5	8		1050.9	4	1098.7	30		1454.8	9	1411.0	8
A′ CH_3 wag		1376.2	4	1335.7	3		1046.2	1	1026.4	4		1367.0	4	1332.0	3
A′ N–Be str.	1082.3	1106.0	76	1092.0	69	1020.8	1043.4	77	1026.9	183	1068.7	1091.5	79	1082.3	74
A′ CH_3 twist		1045.2	29	995.6	32	Covered[c]	875.8	82	858.8	85		1029.1	20	987.3	29
A″ CH_3 twist		988.6	0	953.0	0		780.8	1	752.4	0		978.2	0	944.3	0
A′ C–C str.	831.4	807.0	71	787.4	70		731.5	17	716.3	16	811.6	790.0	67	771.4	66
A′ CCN bend		458.5	26	442.4	22		426.6	19	417.6	20		451.2	25	439.9	24
A″ CNBe oop bend		234.4	1	224.9	0		232.0	0	230.5	0		232.8	1	230.8	0
A′ CNBe ip bend		175.7	4	165.7	4		168.4	3	165.8	3		174.3	4	170.8	4

[a] Frequencies and intensities are in cm^{-1} and km/mol. The computed frequencies are given at the B3LYP–D3/aug–cc–pVTZ level of theory. [b] Observed in a Ne matrix. [c] Absorptions covered by precursor bands. $BeNCCH_3$ has a C_s structure.

Table S3 Observed and Calculated Fundamental Frequencies of $CNBeBeCH_3$ Isotopomers in the Ground $^1A'$ State[a]

Approximate Description	$CNBeBeCH_3$					$CNBeBeCD_3$					$^{13}CNBeBe^{13}CH_3$				
	Obs[b]	Harmonic	Int	Anharmonic	Int	Obs[b]	Harmonic	Int	Anharmonic	Int	Obs[b]	Harmonic	Int	Anharmonic	Int
A′ CH_3 as. str.		3039.3	12	2776.3	33		2243.6	4	2107.4	4		3029.1	13	2765.0	13
A″ CH_3 as. str.		3039.3	12	2724.8	32		2243.6	4	2014.8	4		3029.1	13	2718.6	13
A′ CH_3 s. str.		2983.6	2	2820.1	166		2145.2	0	2050.8	0		2980.1	2	2814.9	187
A′ N–C str.	2111.0	2157.7	458	2120.0	414	2111.9	2157.7	458	2129.7	434	2068.1	2117.7	458	2082.0	417
A″ CH_3 twist		1434.8	3	1288.1	0		1129.8	2	1103.3	1		1432.2	3	1267.3	0
A′ CH_3 twist		1434.8	3	963.9	0		1037.2	3	886.1	0		1432.2	3	946.7	0
A′ CH_3 wag	1226.1	1249.1	48	1281.1	45		1037.2	3	621.1	0		1237.2	44	1279.6	41
A′ Be–Be str.	1105.6	1109.8	44	1083.6	16		831.2	56	601.5	108		1105.9	39	1079.4	13
A′ C–Be str.	Covered[c]	909.5	185	877.7	206	1013.4	1004.5	212	1001.6	131	913.4	902.2	185	870.4	204
A′ CH_3 bend		625.6	16	1242.9	16		520.7	9	724.5	9		621.1	15	1250.9	556
A′ BeBeN bend		326.9	6	526.9	254		311.0	9	518.8	194		326.6	7	532.4	255
A′ Be–Be str		324.4	2	261.6	5		309.3	2	257.4	2		317.1	2	254.0	3
A′ BeNC bend		181.2	1	395.4	1		171.6	0	429.1	24		180.2	1	397.1	11
A′ Be–Be bend		83.0	14	94.3	78		79.5	14	162.6	198		81.5	14	87.5	72

[a] Frequencies and intensities are in cm^{-1} and km/mol. The computed frequencies are given at the B3LYP–D3/aug–cc–pVTZ level of theory. [b] Observed in a Ne matrix. [c] Absorptions covered by precursor bands. $CNBeBeCH_3$ has a C_{3v} structure.

Table S4 Observed and Calculated Fundamental Frequencies of $CNBeCH_3$ Isotopomers in the Ground $^1A_1$ State[a]

Approximate Description	$CNBeCH_3$					$CNBeCD_3$					$^{13}CNBe^{13}CH_3$				
	Obs[b]	Harmonic	Int	Anharmonic	Int	Obs[b]	Harmonic	Int	Anharmonic	Int	Obs[b]	Harmonic	Int	Anharmonic	Int
A′ CH_3 as. str.		3061.0	8	2913.3	11		2260.7	2	2176.1	2		3050.6	9	2903.3	12
A″ CH_3 as. str.		3060.9	8	2911.9	11		2260.5	2	2175.3	2		3050.5	9	2902.2	12
A′ CH_3 s. str.		3001.8	0	2888.2	0		2157.4	9	2088.1	144		2998.3	1	2877.7	0
A′ N−C str.	2100.2	2164.8	436	2134.7	429	2101.4	2165.0	428	2126.8	504	2064.3	2125.0	438	2097.3	421
A″ CH_3 bend		1443.1	1	1421.5	1		1042.1	1	1020.6	1		1440.6	0	1418.4	1
A′ CH_3 bend		1442.7	1	1419.1	1		1042.3	1	1021.8	1		1440.3	0	1416.6	1
A′ CH_3 wag	1237.8	1269.2	160	1237.9	180	1205.0	1217.9	301	1202.9	285	1223.8	1255.5	146	1224.3	168
A′ CBeN as. str.	1162.9	1186.4	147	1166.9	128		951.4	0	931.8	0	1160.5	1183.2	152	1164.5	131
A′ CH_3 twist		695.7	88	676.0	83		576.1	83	565.8	81		690.7	87	671.5	82
A′ CBeN bend	668.3	695.6	88	675.8	86		576.0	83	565.8	82		690.5	87	670.9	85
A′ CBeN s. str.		537.3	4	512.6	3		500.6	3	478.3	3		522.8	3	502.6	3
A′ BeNC ip bend		284.0	18	283.4	19		261.5	9	261.0	10		283.6	18	286.1	18
A″ CeNC oop bend		283.8	18	282.7	19		261.4	9	260.3	9		283.4	18	284.5	19
A′ CBeNC ip bend		126.9	13	119.4	11		121.4	13	121.1	12		124.8	12	126.9	11
A′ CBeNC oop bend		126.6	13	118.2	11		121.2	13	119.7	12		124.6	12	125.2	11

[a] Frequencies and intensities are in cm^{-1} and km/mol. The computed frequencies are given at the B3LYP–D3/aug–cc–pVTZ level of theory. [b] Observed in a Ne matrix. $CNBeCH_3$ has a C_{3v} structure.

Table S5 Observed and Calculated Fundamental Frequencies of $HBeCH_2CN$ Isotopomers in the Ground $^1A'$ State[a]

Approximate Description	$HBeCH_2CN$					DBeCD2CN					$HBe^{13}CH_2{}^{13}CN$				
	Obs[b]	Harmonic	Int	Anharmonic	Int	Obs[b]	Harmonic	Int	Anharmonic	Int	Obs[b]	Harmonic	Int	Anharmonic	Int
A′ CH_2 as. str.		3049.0	0	2892.5	1	2288.3	2325.2	42	2295.5	39		3038.3	0	2883.3	1
A′ CH_2 s. str.		3013.1	3	2881.4	5		2254.2	0	2169.2	0		3007.2	3	2875.6	5
A′ C–N str.		2325.8	39	2291.7	35		2188.2	2	2083.0	2		2270.2	35	2240.3	35
A′ Be–H str.	2141.4	2196.1	159	2133.4	158	1608.3	1654.6	121	1616.0	103	2141.8	2195.9	160	2133.4	157
A′ CH_2 sci.		1423.0	11	1399.6	9		1089.3	12	1063.5	9		1418.2	11	1391.1	11
A″ CH_3 bend		1153.7	7	1124.5	5		992.0	41	971.8	41		1146.8	7	1118.5	6
A′ CH_2 rock		1134.7	63	1100.9	59		887.3	1	873.5	1		1116.9	60	1083.8	36
A′ CH_2 wag		954.0	6	947.3	4		845.4	3	838.6	3		934.3	7	929.7	6
A′ C–C str.		804.3	25	781.3	22		696.3	11	690.1	10		794.6	25	775.5	22
A′ Be–H oop bend	685.9	710.2	139	694.0	137	579.9	596.8	96	587.0	93	680.6	703.2	141	688.5	139
A′ Be–H ip bend		587.3	124	582.0	125		540.2	60	534.8	57		581.7	130	578.5	129
A′ CCN bend		462.6	38	459.0	31		399.1	51	399.1	50		453.7	31	453.4	27
A′ Be–H oop bend		435.5	51	433.2	46		366.4	12	367.9	11		432.1	50	431.6	43
A′ CN bend		365.1	8	360.7	6		293.4	23	293.4	22		359.1	6	360.5	7
A′ CCBe bend		137.4	8	133.3	7		123.6	8	126.1	8		136.4	7	138.9	7

[a] Frequencies and intensities are in cm^{-1} and km/mol. The computed frequencies are given at the B3LYP–D3/aug–cc–pVTZ level of theory. [b] Observed in a Ne matrix. $HBeCH_2CN$ has a C_s structure.

Table S6 Observed and Calculated Fundamental Frequencies of $HBeNCCH_2$ Isotopomers in the Ground $^1A_1$ State[a]

Approximate Description	$HBeNCCH_2$					$DBeNCCD_2$					$HBeN^{13}C^{13}CH_2$				
	Obs[b]	Harmonic	Int	Anharmonic	Int	Obs[b]	Harmonic	Int	Anharmonic	Int	Obs[b]	Harmonic	Int	Anharmonic	Int
A′ CH_2 as. str.		3253.2	2	3105.1	1		2422.3	1	2337.0	1		3239.9	2	3091.5	1
A′ CH_2 s. str.		3167.1	22	3037.8	19		2318.8	249	2238.9	284		3161.2	19	3031.4	15
A′ C−N str.	2147.7	2204.0	1309	2149.5	1769	2114.6	2171.9	1129	2129.2	1025	2075.7	2129.1	966	2133.3	651
A′ Be−H str.		2179.9	287	2130.3	0	1661.8	1688.5	232	1646.3	193	2169.0	2197.2	590	2088.3	823
A′ CH_2 sci		1458.6	1	1430.3	3		1358.4	2	1336.5	2		1436.7	0	1409.6	1
A″ CH_2 sci		1312.3	70	1301.0	78		1008.0	46	1002.8	51		1305.0	64	1292.5	73
A′ CH_2 rock		987.8	1	970.2	1		850.4	2	840.6	1		970.2	1	952.6	1
A′ N−Be str.		848.0	73	830.1	66		773.2	36	769.1	28		838.4	70	820.4	64
A′ CH_2 wag		609.6	107	599.6	102		553.5	19	540.4	12		605.0	104	595.8	101
A′ Be−H ip bend		558.6	213	560.3	210		468.9	32	465.8	128		558.4	214	560.2	211
A′ CCN oop bend.		552.8	35	543.7	25		467.7	132	462.2	45		543.7	152	538.1	205
A′ Be−H oop bend		539.5	132	542.8	143		435.4	123	434.1	117		531.1	17	530.7	4
A′ CCN ip bend		440.5	5	440.6	5		375.2	12	373.2	14		432.8	5	433.4	4
A′ N−Be ip bend		149.0	0	154.0	0		130.9	0	133.9	0		148.1	0	153.2	0
A′ N−Be oop bend		124.3	1	128.3	1		112.9	1	113.9	1		123.6	1	127.4	1

[a] Frequencies and intensities are in cm^{-1} and km/mol. The computed frequencies are given at the B3LYP–D3/aug–cc–pVTZ level of theory. [b] Observed in a Ne matrix. $HBeNCCH_2$ has a C_{2v} structure.

Table S7 Calculated Fundamental Frequencies of the cyclic intermediate in the Ground $^1A_1$ State[a]

Approximate Description	Cyclic intermediate			
	Harmonic	Int	Anharmonic	Int
A″ CH_3 as. str.	3095.5	18	2937.1	20
A′ CH_3 as. str.	3074.0	3	2917.6	3
A′ CH_3 s. str.	3014.2	12	2899.2	12
A′ C–N str.	1609.8	168	1567.6	153
A′ CH_3 bend	1460.7	9	1417.7	8
A″ CH_3 bend	1457.5	46	1429.9	34
A′ CH_3 wag	1384.0	15	1348.0	13
A′ CCN bend	1225.6	4	1195.5	4
A′ CH_3 twist	1095.5	71	1057.5	67
A″ CH_3 twist	985.3	2	956.0	2
A′ CNBeBe str.	890.9	40	875.2	41
A′ C-Be Str.	768.1	66	742.3	64
A′ Be-Be Str.	652.7	20	639.0	21
A′ CNBe ip bend	563.4	63	547.8	62
A′ CCN oop bend	404.0	1	400.0	1
A′ CCN ip bend	305.4	5	301.6	5
A′ CNBe oop bend	193.0	6	192.2	5
A′ CH_3 tort	93.1	1	146.8	0

[a] Frequencies and intensities are in cm^{-1} and km/mol. The computed frequencies are given at the B3LYP–D3/aug–cc–pVTZ level of theory.

Table S8 NBO Analysis of Products Using the B3LYP–D3/aug–cc–pVTZ Level of Theory

Molecule	Bond	WBO	Bond orbitals	Occ
$BeBeNCCH_3$	C–N σ bond	C–N: 1.947 N–Be: 0.558 Be–Be: 0.791	38.73% C($s^{0.42}p^{0.58}$)+61.27% N($s^{0.57}p^{0.43}$)	1.99
	C–N π bond		32.54% C($p^{1.00}$)+67.46% N($p^{1.00}$)	1.97
	N–Be π bond		77.42% N($s^{0.01}p^{0.99}$)+22.58% Be($s^{0.42}p^{0.57}$)	1.66
	Be–Be σ bond		46.87% Be($s^{0.35}p^{0.65}$)+53.13% Be($s^{0.57}p^{0.43}$)	1.60
$BeNCCH_3$	C–N σ bond α-spin	C–N: 1.951 N–Be: 0.809	37.94% C($s^{0.38}p^{0.62}$)+62.06% N($s^{0.56}p^{0.44}$)	1.00
	C–N σ bond β-spin		38.00% C($s^{0.41}p^{0.59}$)+62.00% N($s^{0.58}p^{0.42}$)	1.00
	C–N π bond α-spin		32.02% C($p^{1.00}$)+67.98% N($p^{1.00}$)	0.98
	C–N π bond β-spin		31.91% C($s^{0.07}p^{0.92}$)+68.09% N($p^{1.00}$)	0.98
	N–Be π bond α-spin		88.00% N($s^{0.01}p^{0.99}$)+12.00% Be($s^{0.01}p^{0.99}$)	0.97
	N–Be π bond β-spin		98.26% N($p^{1.00}$)+1.74% Be($p^{0.98}d^{0.02}$)	0.71
$CNBeBeCH_3$	C–Be σ bond	C–Be: 0.976 Be–Be: 0.966 N–Be: 0.977 N–C: 2.966	82.79% C($s^{0.33}p^{0.67}$)+17.21% Be($s^{0.49}p^{0.51}$)	1.98
	Be–Be σ bond		47.65% Be($s^{0.50}p^{0.50}$)+52.35% Be($s^{0.61}p^{0.39}$)	1.94
	N–Be σ bond		11.30% Be($s^{0.39}p^{0.61}$)+88.70% N($s^{0.50}p^{0.50}$)	1.99
	N–C σ bond		65.81% N($s^{0.51}p^{0.49}$)+34.19% C($s^{0.33}p^{0.67}$)	2.00
	N–C π bond		74.44% N($p^{1.00}$)+25.56% C($p^{1.00}$)	1.97
	N–C π bond		74.44% N($p^{1.00}$)+25.56% C($p^{1.00}$)	1.97
$CNBeCH_3$	C–Be σ bond	C–Be: 0.993 N–Be: 0.983 N–C: 2.964	81.06% C($s^{0.30}p^{0.70}$)+18.94% Be($s^{0.60}p^{0.40}$)	1.99
	N–Be σ bond		11.57% Be($s^{0.40}p^{0.60}$)+88.43% N($s^{0.50}p^{0.50}$)	1.99
	N–C σ bond		66.20% N($s^{0.50}p^{0.49}$)+33.80% C($s^{0.32}p^{0.67}$)	2.00
	N–C π bond		74.41% N($p^{1.00}$)+25.59% C($p^{1.00}$)	1.97
	N–C π bond		74.41% N($p^{1.00}$)+25.59% C($p^{1.00}$)	1.97
$HBeCH_2CN$	C–C σ bond	C–C: 0.985 C–Be: 0.950 C–N: 2.923	50.93% C($s^{0.26}p^{0.74}$)+49.07% C($s^{0.53}p^{0.47}$)	1.99
	C–Be σ bond		83.99% C($s^{0.26}p^{0.74}$)+16.01% Be($s^{0.50}p^{0.50}$)	1.92
	C–N σ bond		42.85% C($s^{0.47}p^{0.53}$)+57.15% N($s^{0.47}p^{0.52}d^{0.01}$)	2.00
	C–N π bond		44.21% C($p^{1.00}$)+55.79% N($p^{1.00}$)	1.99
	C–N π bond		45.69% C($p^{1.00}$)+54.31% N($p^{1.00}$)	1.98
$HBeNCCH_2$	C–C σ bond	C–C: 1.825 N–Be: 0.986 C–N: 1.924	48.28% C($s^{0.36}p^{0.64}$)+51.72% C($s^{0.54}p^{0.46}$)	1.99
	C–C π bond		60.97% C($p^{1.00}$)+39.03% C($p^{1.00}$)	1.97
	N–Be σ bond		88.01% N($s^{0.55}p^{0.45}$)+11.99% Be($s^{0.42}p^{0.58}$)	1.98
	C–N σ bond		59.78% N($s^{0.45}p^{0.55}$)+40.22% C($s^{0.46}p^{0.54}$)	1.99
	C–N π bond		67.27% N($p^{1.00}$)+32.73% C($p^{1.00}$)	1.96

Table S9 EDA-NOCV Results at the B3LYP/TZ2P/ZORA Level of Theory for $CNBeBeCH_3$.

Energy term[a]	$CNBe + BeCH_3$
ΔE_{int}	-73.72
ΔE_{Steric}	-32.11
ΔE_{pauli}	33.43
ΔE_{elstat}[b]	-65.54(61.2 %)
ΔE_{orb}[b]	-41.61(38.8 %)
$\Delta E_{orb}(\sigma)$[c]	-41.13(98.8 %)
ΔE_{rest}	-0.48

[a] Energy Values are Given in kcal/mol. [b] The percentage contribution to the total attractive interactions ($\Delta E_{elstat} + \Delta E_{orb} + \Delta E_{dis}$) is given in parentheses. [c] The percentage contribution to the total orbital interactions is given in parentheses.

Table S10 AIM Analysis of BeN, BeC and BeBe bonds in Products.

	$BeBeNCCH_3$	$CNBeBeCH_3$	Be_2	$BeNCCH_3$	$CNBeCH_3$	$HBeCH_2CN$	CH_2CNBeH
Be–N							
$\nabla^2\rho_{cp}$	0.6479	0.6078		0.7387	0.6073		0.7175
E(r)	-0.2494	-0.3663		-0.3440	-0.3788		-0.3909
Be–C							
$\nabla^2\rho_{cp}$		0.2116			0.2164	0.1930	
E(r)		-0.5694			-0.5713	-0.5182	
Be–Be							
$\nabla^2\rho_{cp}$	-0.1176	-0.1079	-0.2939				
E(r)	-0.3154	-0.3997	-0.8727				
